# Exploring the Impact of Artificial Intelligence on the Creativity Perception of Music Practitioners

**DOI:** 10.3390/jintelligence13040047

**Published:** 2025-04-15

**Authors:** Haixia Ma, Yan Zhang, Xin Shan, Xiaoxi Hu

**Affiliations:** 1 College of Music, Kookmin University, Seoul 02707, Republic of Korea; mahaixia814@kookmin.ac.kr; 2 The Graduate School Arts & Culture, Sangmyung University, Seoul 03016, Republic of Korea; 201932049@sangmyung.kr; 3 State Key Laboratory of Advanced Rail Autonomous Operation, Beijing Jiaotong University, Beijing 100044, China

**Keywords:** artificial intelligence, music composition, empirical analysis, music practitioners

## Abstract

This study investigates the relationship between artificial intelligence (AI) tools and the creative abilities of music practitioners within the context of globalization and technological advancements that are transforming creative industries. Through a quantitative analysis, the study assesses how AI tool usage influences creative output. By surveying music practitioners from diverse backgrounds, it captures their experiences and perceptions of AI technologies in music creation. Grounded in cognitive science and diffusion of innovation theories, the research also empirically examines the relationship between AI technology acceptance and creativity perception, while considering the role of socioeconomic factors. Regression analysis was used to explore the relationships between key variables, ensuring robust and reliable results. The results suggest that AI technology acceptance is significantly correlated with creative performance, particularly among individuals with formal music education and experience using music composition software. However, socioeconomic factors such as age, gender, and professional background also influence how extensively AI is utilized in the creative process. These findings provide new insights into AI’s role in creative industries and offer data to inform music education and technology training policies.

## 1. Introduction

In today’s era of accelerated globalization and ongoing technological advancements, artificial intelligence (AI) has emerged as a key technology driving innovation across various industries. AI applications are widespread, ranging from automated diagnostics in healthcare to risk assessment in financial services, bringing new phases of development to different sectors (Chang et al. 2021; Zhang et al. 2019; Siphocly et al. 2021; Sturm et al. 2018; Scurto and Chemla-Romeu-Santos 2021). The music industry, as a vital component of the cultural and creative industries, is similarly undergoing profound transformations due to AI. The integration of AI technologies has not only altered the music production process but has also expanded the creative capabilities and potential of music practitioners (Michałko et al. 2022, 2024; Campo et al. 2023; Zulić 2019). Despite the increasing use of AI, systematic research examining its actual influence on music compositional abilities remains limited (Tigre Moura et al. 2023; Anantrasirichai and Bull 2022). Additionally, a recent study focusing on AI-based music composition found that reflection, a crucial element in creative practice, has multiple underexplored aspects when dealing with AI generated content (AIGC). The research showed that composers often reflect on the future directions of their music while using AIGC tools, highlighting the need for more in-depth exploration in this area (Ford et al. 2024).

This study uses a survey to examine how AI tools for creating musical scores—such as composition software, editing programs, and synthesis systems—help musicians improve their work. By focusing on tools that generate melodies, harmonies, and rhythms based on music theory, the research measures how widely these technologies are used today and identifies their benefits for creativity and efficiency. The results aim to offer practical insights for developing better AI tools and supporting innovation in music creation.

## 2. Hypothesis

This study seeks to incorporate the acceptance of AI technology into the analytical framework of music creation ability and to explore its impact on the effectiveness of music creation. Based on existing research, this study proposes the following hypotheses (see Table 1).

AI technology provides music practitioners with a range of innovative tools and methods, including music editing software, synthesis tools, and intelligent composition programs (Anantrasirichai and Bull 2022; Budagyan and Zaytseva 2020). A high level of acceptance of these technologies suggests that music practitioners are willing to adopt them and are consequently able to more effectively utilize these tools to produce more complex and innovative musical works (Budagyan and Zaytseva 2020). As a result, we expect that individuals with a higher level of acceptance of AI technology will demonstrate better musical compositional performance.

**Hypothesis** **1:** *A high level of acceptance of AI technology among music practitioners significantly enhances music practitioners’ compositional effectiveness.*

According to cognitive science, younger professionals possess more plastic cognitive structures, making it easier for them to adopt and learn new technologies (Solyst et al. 2023; Almaraz-López et al. 2023; Sun 2020). Additionally, diffusion of innovation theory suggests that young professionals often act as early adopters, with their openness and technical backgrounds driving quicker adoption of AI (Almaraz-López et al. 2023). Younger music practitioners tend to be more open to new technologies and adapt more quickly to them, enabling them to grasp and apply AI technologies to music creation more effectively (Sun 2020). This adaptability allows younger practitioners to take advantage of AI technology more efficiently in their music compositions, often resulting in more innovative and complex works compared to their older counterparts.

**Hypothesis** **2:** *Among music practitioners of different ages, younger music practitioners’ acceptance of AI technology plays a more significant role in enhancing the effectiveness of music creation.*

Studies have shown that women tend to show higher levels of adaptability and innovation when adopting new technologies (Born and Devine 2016; De Araújo et al. 2001; Nenci 2013). Research indicates that women’s involvement in technology not only drives innovation but also addresses gender inequalities. For instance, women play a significant role in shaping technological advancements through their daily interactions with products, inspiring practical innovations (De Araújo et al. 2001). In the field of music composition, this tendency may lead female practitioners to explore the creative potential of AI technologies more actively, thereby demonstrating higher levels of creativity and technical proficiency in their compositions. Women’s expertise in synthesizing data and using imaginative judgment, often described as ‘web thinking’, further contributes significantly to technological innovation (Nenci 2013). This adaptability and innovation reinforce women’s crucial role in shaping technological progress across various domains.

**Hypothesis** **3:** *Among music practitioners of different genders, women’s acceptance of AI technology plays a more significant role in enhancing the effectiveness of their music creation.*

## 3. Research Design and Data Analysis

### 3.1. Data Sources

In April 2024, we conducted a questionnaire survey, combined with statistical analysis, targeting music practitioners across China. A total of 800 questionnaires were distributed, including 300 paper-based and 500 online questionnaires. To ensure distribution of the surveys, we shared the questionnaire link through email and WeChat in music colleges, music schools, and relevant industry organizations nationwide, inviting participants to fill it out. Additionally, to incentivize participation, we incorporated the red packet mechanism—a traditional Chinese custom of gifting money in envelopes symbolizing good fortune—by randomly awarding small monetary rewards to selected respondents through direct transfers.

To encourage participants to complete the survey, we offered a random electronic red packet of cash ranging from 1 to 100 RMB to all respondents, adding an element of fun to their participation and expressing our gratitude for their valuable time. Participants were informed that their feedback would be used to link music creation and the application of AI technology, and they were assured that all data would be kept strictly confidential and used solely for academic research.

Ultimately, 713 questionnaires were retrieved, resulting in a completion rate of 89.13%. After data cleaning, 663 questionnaires were deemed valid, yielding a validity rate of 93.99%. In this study, valid questionnaires refer to those without missing data.

The demographic breakdown of the valid responses indicated that 7.08% of the respondents were under 18 years of age, 19.00% were aged 18–24, 35.89% were aged 25–34, 18.85% were aged 35–44, and 19.15% were 45 years of age or older. In terms of gender, males accounted for 54.90% of the respondents, while females accounted for 45.09%. In terms of musical background, 47.21% of the respondents identified as amateur music practitioners, 33.48% as semi-professional practitioners, and 19.31% as professional practitioners.

**Figure 1:**
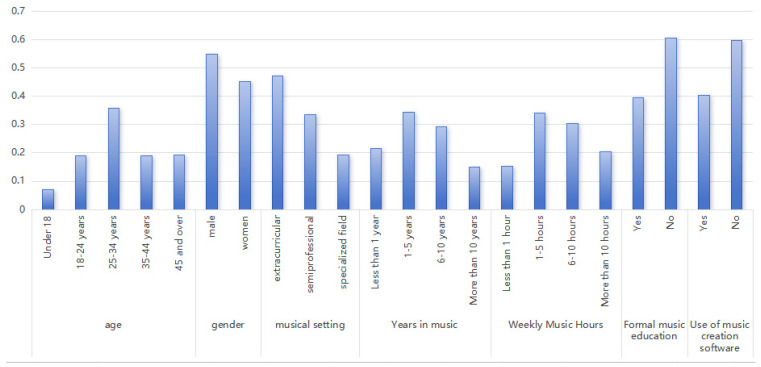
Statistics on the composition of the valid sample.

In terms of musical experience, 21.42% of respondents had been engaged in music activities for less than 1 year, 34.24% for 1–5 years, 29.26% for 6–10 years, and 15.08% for more than 10 years. In terms of weekly time spent on music activities, 15.23% of respondents engaged in less than 1 h per week, 34.09% spent 1–5 h, 30.32% spent 6–10 h, and 20.36% spent more than 10 h per week. In terms of music educational background, 39.37% of respondents had received formal music education, while 60.63% had not. Finally, 40.42% of respondents reported using some form of music composition software or tools, while 59.58% did not. See Figure 1 and Table A1 for details.

### 3.2. Definition of Variables

The questionnaire design for this study draws on recent research in the field of music composition and artificial intelligence, with a particular focus on the application of AI music tools. The questionnaire was divided into three dimensions: basic information, technology acceptance, and creative influence.

The first dimension collected participants’ personal information, including their age group, gender, musical background, number of years engaged in music activities, main instrument or software used, weekly time engaged in music activities, whether they had received formal music education, the contexts in which they typically used music creation tools, and their main motivations for creating music.

The second dimension measured participants’ attitudes toward the use of AI in music composition. This included overall attitudes toward AI music tools, perceptions of AI assistance in composition, willingness to experiment with new AI music tools, satisfaction with the user interface of AI music tools, need for technical support, the perceived capability of AI to solve technological problems, the role of AI in music theory education, and expectations for the future development of AI music tools.

The third dimension explored perceived changes resulting from the use of AI music tools, including impacts on composition speed, composition quality, variety of musical styles, discovery of new musical inspirations, attention to technical details, collaboration with other music creators, freedom of creative expression, understanding of music theory and compositional techniques, originality, and error reduction.

The basic information dimension collected data on participants’ age, gender and other individual attributes to analyze differences in AI acceptance among different age and gender groups and their impact on music creation, aiming to prove Hypotheses 2 and 3. The technology acceptance dimension measured participants’ attitudes towards AI application in music composition to assess their acceptance of AI technology, serving to prove Hypothesis 1. The creative influence dimension explored perceived changes in music creation after using AI music tools, which can prove all three hypotheses. Through systematic analysis of data from these three dimensions, hypotheses about the relationship between AI acceptance and key elements of music creation can be verified. A five-point Likert scale was used, with “1” representing complete disagreement or unfamiliarity and “5” representing complete agreement or familiarity; specific questions are shown in Table 2.

## 4. Empirical Results and Analysis

### 4.1. Validity Testing of Questionnaires

SPSS 20.0 was used to validate the reliability of the data for all variables covered in the study.

#### 4.1.1. Normal Distribution Test

**Figure 2:**
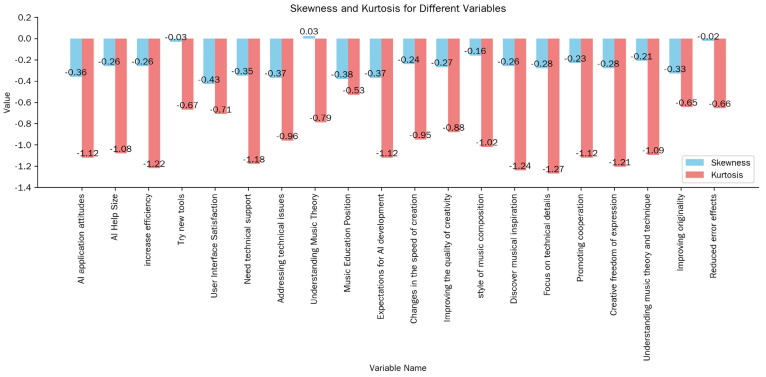
Descriptive statistics for each item.

The fundamental assumption for statistical analysis of questionnaire data is that the data follow a normal distribution. To verify this, the questionnaire items were analyzed for mean, standard deviation, skewness, and kurtosis. It is generally believed that if the absolute value of the skewness coefficient is less than 3 and the absolute value of the kurtosis coefficient is less than 10, the data can be considered to be normally distributed. The specific test results are presented in Table 3 and Figure 2.

As shown in Table 3, the absolute value of the skewness coefficient for each item is less than 1, and the absolute value of the kurtosis coefficient is less than 2. These results indicate that the data from the formal questionnaire conform to a normal distribution.

#### 4.1.2. Confirmatory Factor Analysis

After conducting reliability and validity tests, a confirmatory factor analysis (CFA) model was employed to determine whether the questionnaire’s sample data and the hypothesized model structure were consistent with the actual situation. Based on the fit indices presented in Table 4, the constructed model demonstrates excellent fit, possessing strong explanatory and predictive capabilities. The Goodness of Fit Index (GFI) is 0.976, the Root Mean Square Error of Approximation (RMSEA) is 0.027, and the Root Mean Square Residual (RMR) is 0.036. These indices either exceed or meet the respective criteria, showing a high degree of concordance between the model and the actual data.

Furthermore, the Comparative Fit Index (CFI) is 0.992, the Normed Fit Index (NFI) is 0.976, and the Non-Normed Fit Index (NNFI) is 0.991. These high values further confirm the model’s excellent fit across different dimensions.

In summary, considering all the fit indices, the model exhibits a very high degree of correspondence with the actual data, has a reasonable structure, and possesses good explanatory and predictive capabilities, making it suitable for the research purpose.

#### 4.1.3. Reliability Analysis

Cronbach’s alpha test was performed on the collected data using SPSS 20.0, and the results are presented in Table 5. As shown in the table, Cronbach’s alpha coefficients for each scale are greater than 0.8, indicating a high level of reliability and confirming that the data are highly reliable.

#### 4.1.4. Validity Analysis

The primary objective of structural validity is to assess the validity of the questionnaire’s structure. Key evaluation metrics include the KMO test, Bartlett’s test of Sphericity, cumulative contribution rate, and factor loadings. The KMO test and Bartlett’s test determine the suitability of the questionnaire data for factor analysis. The cumulative contribution rate represents the degree of cumulative validity of the public factor on the scale, while factor loadings indicate the correlation between the original variables and the common factors.

The analysis results show that the KMO metric value for the Perceived Environmental Governance Effectiveness Scale is 0.97, and Bartlett’s test of Sphericity yielded an approximate chi-square value of 11,876.217 (*p* < 0.001). These results suggest that the data are suitable for factor analysis. The eigenvalue criterion (greater than 1) was used to extract factors, resulting in the extraction of two main components. As shown in Table 6, the proportion of cumulative explained variance was 55.79%. The factors associated with 20 items aligned with the initial dimensions, and no cross-factor loading was observed, indicating that the scale’s structural validity is relatively high.

### 4.2. Bivariate Analyses

To analyze the relationship between AI technology acceptance and the effectiveness of music creation, a bivariate analysis was first conducted. The results of the correlation matrix are presented in Table 7.

The correlation analysis shows a significant positive relationship between AI technology acceptance and the effectiveness of music creation (r = 0.6311, *p* < 0.001). Additionally, there is a significant negative correlation between age and AI technology acceptance. These results suggest that younger participants are more likely to accept AI technology. To further verify the relationships among these variables and explore their connections, regression analysis was subsequently conducted. The purpose of the regression analysis was to predict the effectiveness of music creation (as the dependent variable) and examine the impact of AI technology acceptance, age, gender, musical background, and time spent on music activities on it.

### 4.3. Regression Analysis

The results of the regression analysis indicate that AI technology acceptance is the most significant predictor of creativity perception among music practitioners (β = 0.60, *p* < 0.001). This strong positive relationship suggests that participants who are more accepting of AI technologies report significantly higher levels of creativity in their music creation processes. This finding underscores the importance of technological integration in enhancing creative capabilities within the field of music (see Table 8).

In terms of control variables, age negatively correlates with creativity perception (β = −0.07, *p* < 0.001), indicating that older participants tend to report lower creativity levels, potentially due to generational differences in technology use. The gender variable showed no significant correlation (β = 0.01, *p* = 0.12). A positive correlation was found for musical setting (β = 0.13, *p* < 0.001), suggesting that a supportive environment is linked to creativity. Years of activity negatively correlated with creativity (β = −0.05, *p* < 0.05), possibly reflecting creative fatigue, while activity time positively influenced perception (β = 0.05, *p* < 0.05), indicating that more engagement leads to greater creativity. Formal education (β = 0.15, *p* < 0.01) and music software use (β = 0.14, *p* < 0.01) are also positive predictors, emphasizing the importance of structured learning and technological tools in fostering creativity.

### 4.4. Heterogeneity Analysis

To examine whether the relationship between AI technology acceptance and music practitioners’ attitudes toward creativity varies across different demographic and professional groups, we conducted subgroup analyses using ordinary least squares (OLS) regression models. These analyses were performed separately based on age, specialization level, years of musical activity, experience with music composition tools, and gender. Control variables included age (when not the grouping variable), gender, musical setting, years of activity (when not the grouping variable), activity time, formal education, and use of music software.

The group regression analysis in Table 9 indicates that there is a significant age heterogeneity in the impact of AI technology acceptance on creativity perception. Through the Chow test, significant differences in regression coefficients among different age groups were found to be statistically significant. Although all age groups show a significant positive association, with standardized regression coefficients ranging from 0.48 to 0.72 and all being significant at the 1% level, the intensity of the effect presents a typical bimodal distribution pattern. Specifically, the age group of 25–34 years old and the group of 45 years old and above exhibit the highest effect values of 0.72 and 0.70, respectively, which are 50.0% and 45.8% higher than the baseline value of 0.48 of the group under 18 years old. However, in the middle-aged group of 35–44 years old, the effect size significantly drops back to 0.51, forming a trough in the age-effect curve, with a drop of 0.21. The results reveal that age moderates the relationship between AI technology acceptance and attitudes towards creative outcomes, although not as initially hypothesized. Therefore, Hypothesis 2 was not supported.

The regression analysis in Table 10 clearly confirms that gender has a significant moderating effect on the relationship between the acceptance of AI technology and the perception of creativity. According to the data, the strength of the moderating effect exhibited by female music practitioners is significantly higher than that of their male counterparts. The standardized regression coefficients are 0.65 for females and 0.55 for males. This indicates that when the acceptance of AI technology increases by one unit, the relative increase in the perception of creativity among females reaches 18.2%. This result strongly supports the moderating effect model proposed in Hypothesis 3. It is worth noting that the variable of formal education shows an obvious characteristic of gender heterogeneity. In the male group, the standardized effect size of formal education is significant, with a value of 0.25. However, in the female group, no significant statistical association between formal education and related variables has been observed.

## 5. Discussion and Conclusions

Our study found that music practitioners who reported higher acceptance of AI technology also reported higher levels of creative output. This aligns with Chubb et al. (2022), who observed a positive correlation between technology acceptance and self-reported creativity across various domains. According to cognitive load theory (Hornay 2021; Chen et al. 2018), AI may help practitioners reduce cognitive demands by handling complex tasks, allowing them to focus more on creative exploration. Participants perceived that AI assists in providing complex musical elements and materials, enhancing efficiency and enabling them to transcend traditional compositional limitations.

Participants with formal music education and experience using music composition software reported superior creative abilities, consistent with Zhang et al. (2021), who emphasized the role of educational background and technological proficiency in artistic performance. This suggests that education and experience enhance individuals’ ability to understand and apply AI technologies effectively. The use of music creation software significantly influenced participants’ perceptions of AI’s effectiveness. Those with software experience reported stronger positive correlations, highlighting the importance of technological proficiency (Geerts et al. 2023). Amateur practitioners showed smaller effects, possibly due to less systematic training. Unlike prior research focusing on AI development and application (Sturm et al. 2019), our study used self-reported data to reveal that AI technology enhances perceived creative efficiency and freedom among music practitioners. We also found generational differences in AI adoption, with younger creators more inclined to embrace AI (Kehagia and Moriaty 2023), offering new perspectives for music education and technology training policies.

Our heterogeneity analysis revealed that age moderates the relationship between AI technology acceptance and self-reported creative output. Specifically, practitioners aged 25–34 and those 45 and over reported stronger positive correlations, possibly due to their accumulated experience and understanding of the profession (Lee 2022). Younger practitioners may lack the domain-specific expertise to fully leverage AI technologies (Charness and Boot 2009). This indicates a need for age-specific approaches in music education to provide appropriate technological resources.

Gender differences were also noted, with female participants reporting greater creative effectiveness when using AI technologies. This may relate to a stronger inclination to enhance emotional expression in music, aligning with Born and Devine’s (2016) findings. Considering gender differences in emotional intelligence (Papoutsi et al. 2022; Donges et al. 2012) underscores the importance of equitable music technology training.

This study has limitations. The reliance on self-reported data may introduce response biases and may not fully represent practitioners in different contexts. The cross-sectional design prevents inferences between AI technology acceptance and creative abilities. Future research should consider longitudinal studies and include diverse geographic and cultural contexts to enhance generalizability.

Our findings emphasize that acceptance of AI technology is associated with enhanced self-reported creative abilities among music practitioners, influenced by factors such as age, gender, and professional experience. These insights highlight the need to strengthen music technology education to foster creativity, which contributes to individual artistic growth and broader cultural innovation.

## Figures and Tables

**Table 1:** Research hypotheses.

Hypothesis Number	Hypothesis Statement	Supporting Information
H1	A high level of acceptance of AI technology among music practitioners significantly correlates with their compositional engagement.	AI tools like music editing software, synthesis tools, and intelligent composition programs contribute to the interplay of effectiveness and innovation in musical productions (Anantrasirichai and Bull 2022; Budagyan and Zaytseva 2020).
H2	Among music practitioners of different ages, younger practitioners’ acceptance of AI technology plays a more significant role in enhancing the interactivity of music creation.	Younger professionals have adaptable cognitive structures and are early adopters, facilitating quicker integration of AI tools (Solyst et al. 2023; Almaraz-López et al. 2023; Sun 2020).
H3	Among music practitioners of different genders, women’s acceptance of AI technology plays a more significant role in enhancing the effectiveness of their music creation.	Women show stronger adaptability and innovation when adopting new technologies. In the field of music composition, their “web thinking” enables female practitioners to make better use of AI technology, tap into creative potential, and enhance the creativity and technical level of their works (Born and Devine 2016; De Araújo et al. 2001; Nenci 2013).

**Table 2:** Variable definitions and assignments.

No.	Category	Question	Encodings
1	Basics	What is your age group?	Under 18 = 1, 18–24 = 2, 25–34 = 3, 35–44 = 4, 45+ = 5
2		What is your gender?	Male = 1, Female = 2
3		What is your musical background?	Amateur = 1, Semi = 2, Professional = 3
4		How many years have you been engaged in musical activities?	<1 year = 1, 1–5 = 2, 6–10 = 3, >10 = 4
5		How much time do you spend on average per week engaged in musical activities?	<1 h = 1, 1–5 = 2, 6–10 = 3, >10 = 4
6		Have you received formal music education?	No = 1, Yes = 2
7		Have you used any type of music composition software or tools before?	No = 1, Yes = 2
8	AI technology acceptance	What is your attitude towards the application of AI in music composition?	Very positive = 5, Positive = 4, Neutral = 3, Negative = 2, Very negative = 1
9		How much do you think AI music tools can help with composition?	Very large = 5, Large = 4, Average = 3, Small = 2, Very small = 1
10		Do you think AI music tools can improve the efficiency of music composition?	Strongly agree = 5, Agree = 4, Neutral = 3, Disagree = 2, Strongly disagree = 1
11		Are you willing to try new AI music composition tools?	Very willing = 5, Willing = 4, Neutral = 3, Unwilling = 2, Very unwilling = 1
12		How user-friendly do you think the user interface design of AI music tools is for the composition experience?	Very satisfied = 5, Satisfied = 4, Neutral = 3, Dissatisfied = 2, Very dissatisfied = 1
13		How crucial do you think the technical support provided by AI music tools is for the realization of their composition value?	Very satisfied = 5, Satisfied = 4, Fair = 3, Not satisfied = 2, Unsatisfied = 1
14		Do you think AI can help you solve the technical problems encountered in music composition?	Strongly agree = 5, Agree = 4, Neutral = 3, Disagree = 2, Strongly disagree = 1
15		Do you think AI music tools are helpful for the understanding of music theory?	Strongly agree = 5, Agree = 4, Neutral = 3, Disagree = 2, Strongly disagree = 1
16		What position do you think AI music tools should occupy in music education?	Very important = 5, Important = 4, Fair = 3, Not important = 2, Very unimportant = 1
17		What are your expectations for the future development of AI music tools?	Highly expectant = 5, Expectant = 4, Neutral = 3, Not expectant = 2, Very not expectant = 1
18	Creativity Perception	After using AI music tools, how do you think your composition speed has changed?	Significantly faster = 5, Slightly faster = 4, No change = 3, Slightly slower = 2, Significantly slower = 1
19		Do you think AI tools are helpful for improving the quality of your compositions?	Very helpful = 5, Helpful = 4, Fair = 3, Not helpful = 2, Not helpful at all = 1
20		After using AI tools, has the style of your music works become more diversified?	Strongly agree = 5, Agree = 4, Neutral = 3, Disagree = 2, Strongly disagree = 1
21		After using AI tools, have you discovered new musical inspirations?	Very often = 5, Frequently = 4, Sometimes = 3, Rarely = 2, Never = 1
22		Does the AI tool make you pay more attention to the technical details of music when composing?	Strongly agree = 5, Agree = 4, Neutral = 3, Disagree = 2, Strongly disagree = 1
23		Do you think AI tools have promoted your cooperation with other music creators?	Strongly agree = 5, Agree = 4, Neutral = 3, Disagree = 2, Strongly disagree = 1
24		When using AI tools to compose music, do you feel more free to express your creativity?	Strongly agree = 5, Agree = 4, Neutral = 3, Disagree = 2, Strongly disagree = 1
25		Do you think AI tools have helped you better understand music theory and composition techniques?	Strongly agree = 5, Agree = 4, Neutral = 3, Disagree = 2, Strongly disagree = 1
26		In your opinion, how much impact do AI tools have on improving the originality of music works?	Very large = 5, Large = 4, Average = 3, Small = 2, Very small = 1
27		How effective do you think AI tools are in reducing music composition errors?	Very good = 5, Good = 4, Fair = 3, Poor = 2, Very poor = 1

**Table 3:** Descriptive statistics for each item.

Variable Name	Average Value	(Statistics) Standard Deviation	Skewness	Kurtosis
AI application attitudes	3.3	1.39	−0.36	−1.12
AI help size	3.14	1.32	−0.26	−1.08
Increase efficiency	3.25	1.40	−0.26	−1.22
Try new tools	2.98	1.19	−0.03	−0.67
User interface satisfaction	3.28	1.25	−0.43	−0.71
Need technical support	3.33	1.40	−0.35	−1.18
Addressing technical issues	2.98	1.22	−0.37	−0.96
Understanding music theory	2.93	1.23	0.025	−0.79
Music education position	3.28	1.17	−0.38	−0.53
Expectations for AI development	3.31	1.38	−0.37	−1.12
Changes in the speed of creation	2.96	1.19	−0.24	−0.95
Improving the quality of creativity	3.35	1.25	−0.27	−0.88
Style of music composition	3.12	1.28	−0.16	−1.02
Discover musical inspiration	3.25	1.41	−0.26	−1.24
Focus on technical details	3.29	1.43	−0.28	−1.27
Promoting cooperation	3.637	1.044	−0.23	−1.121
Creative freedom of expression	3.214	1.401	−0.28	−1.205
Understanding music theory and technique	3.084	1.321	−0.211	−1.094
Improving originality	3.238	1.198	−0.33	−0.645
Reduced error effects	2.925	1.187	−0.022	−0.655

**Table 4:** Confirmatory factor analysis results.

Fit Index	Value	Criteria
GFI	0.976	>0.9
RMSEA	0.027	<0.10
RMR	0.036	<0.05
CFI	0.992	>0.9
NFI	0.976	>0.9
NNFI	0.991	>0.9

**Table 5:** Cronbach’s alpha coefficients for the scale.

Variant	Quantities	Cronbach’s Alpha Coefficient	Confidence Level
AI technology acceptance	10	0.94	high reliability
Creativity perception	10	0.94	high reliability

**Table 6:** Exploratory factor analysis of the scale.

	Factor 1	Factor 2	Commonality (Common Factor Variance)
AI application attitudes		0.76	0.71
AI help size		0.75	0.7
Increase efficiency		0.78	0.74
Try new tools		0.72	0.59
User interface satisfaction		0.70	0.61
Need technical support		0.79	0.75
Addressing technical issues		0.72	0.66
Understanding music theory		0.72	0.60
Music education position		0.70	0.60
Expectations for AI development		0.78	0.73
Changes in the speed of creation	0.76		0.65
Improving the quality of creativity	0.74		0.66
Style of music composition	0.75		0.67
Discover musical inspiration	0.80		0.73
Focus on technical details	0.81		0.76
Promoting cooperation	0.81		0.74
Creative freedom of expression	0.78		0.72
Understanding music theory and technique	0.77		0.68
Improving originality	0.68		0.59
Reduced error effects	0.74		0.59
	28.39%	55.79%	
KMO value	0.97		
Bartlett’s test of Sphericity	approximate chi-square (math.)	11,876.21	
	*p*	0.000 ***	

Note: *** represent significance levels of *p* < 0.001.

**Table 7:** Correlation matrix of variables.

Variables	Creativity Perception	AI Technology Acceptance	Age	Gender	Musical Setting	Years of Activity	Activity Time	Formal Education	Music Software
Creativity perception	1.000								
AI technology acceptance	0.631 ***	1.000							
Age	−0.280 ***	−0.311 ***	1.000						
Gender	0.038	−0.064 *	0.034	1.000					
Musical setting	0.007	−0.138 ***	0.088 **	0.045	1.000				
Years of activity	−0.074 *	−0.018	0.066 *	−0.014	−0.057	1.000			
Activity time	0.096 **	0.067 *	−0.052	−0.027	−0.010	0.008	1.000		
Formal education	0.114 ***	0.062	−0.122 ***	−0.039	−0.132 ***	−0.015	0.015	1.000	
Music software	0.095 **	0.020	−0.018	−0.013	0.093 **	0.000	0.002	0.085 **	1.000

Note: *, **, and *** represent significance levels of *p* < 0.05, *p* < 0.01, and *p* < 0.001, respectively.

**Table 8:** Relationship between AI technology acceptance and music practitioners’ creativity perception.

Variant	Mold	
	β	t
Age	−0.07 ***	−2.66
Gender	0.01	0.12
Musical setting	0.13 ***	3.28
Years of activity	−0.05 *	−1.73
Activity time	0.05 *	1.73
Formal education	0.15 **	2.42
Music software	0.14 **	2.21
AI technology acceptance	0.60 ***	19.44
F	62.06 *	
Adjustment of R^2^	0.42	

Note: *, **, and *** represent significance levels of *p* < 0.05, *p* < 0.01, and *p* < 0.001, respectively.

**Table 9:** Impact of AI technology acceptance on music practitioners’ creativity perception (by age).

	(1) Creativity Perception (β)	(2) Creativity Perception (β)	(3) Creativity Perception (β)	(4) Creativity Perception (β)	(5) Creativity Perception (β)
Age	Under 18	18–24 years	25–34 years	35–44 years	45 and over
AI technology acceptance	0.48 ***	0.53 ***	0.72 ***	0.51 ***	0.70 ***
	(3.04)	(10.37)	(10.26)	(6.50)	(11.23)
Gender	−0.03	−0.01	−0.07	0.05	0.09
	(−0.10)	(−0.07)	(−0.66)	(0.33)	(0.67)
Musical setting	0.01	0.21 ***	0.13 *	0.01	0.17 *
	(0.05)	(2.93)	(1.66)	(0.08)	(1.81)
Years of activity	−0.07	−0.05	−0.05	−0.15 *	0.01
	(−0.51)	(−0.82)	(−0.96)	(−1.85)	(0.08)
Activity time	0.11	−0.01	−0.02	0.19 **	0.07
	(0.76)	(−0.25)	(−0.30)	(2.27)	(1.05)
Formal education	0.10	0.06	−0.01	0.39 **	0.23 *
	(0.34)	(0.57)	(−0.00)	(2.35)	(1.74)
Music software	0.30	0.11	0.13	0.13	0.14
	(1.00)	(0.95)	(1.13)	(0.75)	(1.03)
Constant	0.91	1.17 ***	0.85 *	0.43	−0.36
	(0.66)	(2.84)	(1.81)	(0.70)	(−0.73)
N	47	238	126	125	127
F	1.49	16.4	17.58	10.26	19.93
R^2^	0.21	0.33	0.51	0.380	0.54
Between-group coefficient difference *p*-value	0.042 **				

Note: *, **, and *** represent significance levels of *p* < 0.05, *p* < 0.01, and *p* < 0.001, respectively. The *p*-values for coefficient differences are calculated based on the estimation results of the seemingly unrelated regression model.

**Table 10:** The correlation of AI technology acceptance with music practitioners’ creativity perception (by gender).

	(1) Creativity Perception (β)	(2) Creativity Perception (β)
Gender	male	female
AI technology acceptance	0.55 ***	0.65 ***
	(12.82)	(14.75)
Age	−0.09 **	−0.04
	(−2.41)	(−1.07)
Musical setting	0.09	0.19 ***
	(1.61)	(3.15)
Years of activity	−0.07	−0.05
	(−1.63)	(−1.10)
Activity time	0.05	0.06
	(1.11)	(1.25)
Formal education	0.25 ***	0.05
	(2.73)	(0.52)
Music software	0.12	0.15 *
	(1.39)	(1.69)
Constant	1.03 ***	0.59 *
	(2.82)	(1.75)
N	364	299
F	32.35	40.47
R^2^	0.39	0.49
Between-group coefficient difference *p*-value	0.072 *	

Note: *, **, and *** represent significance levels of *p* < 0.05, *p* < 0.01, and *p* < 0.001, respectively. The *p*-values for coefficient differences are calculated based on the estimation results of the Chow test for the interaction model.

## Data Availability

General correspondence and requests for source data and materials should be addressed to Haixia Ma. Requests for access to data should be addressed to mahaixia814@kookmin.ac.kr.

## Appendix A

**Table A1:** Statistics on the composition of the valid sample.

Variable	Options (as in Computer Software Settings)	Frequency	Percentage (%)
Age	Under 18	47	7.08
	18–24 years	126	19.00
	25–34 years	238	35.89
	35–44 years	125	18.85
	45 and over	127	19.15
Gender	Male	364	54.90
	Female	299	45.09
Musical setting	Extracurricular	313	47.21
	Semi-professional	222	33.48
	Specialized field	128	19.31
Years in music	Less than 1 year	142	21.42
	1–5 years	227	34.24
	6–10 years	194	29.26
	More than 10 years	100	15.08
Weekly music hours	Less than 1 h	101	15.23
	1–5 h	226	34.09
	6–10 h	201	30.32
	More than 10 h	135	20.36
Formal music education	Yes	261	39.37
	No	402	60.63
Use of music creation software	Yes	268	40.42
	No	395	59.58
